# Blood pressure improvement after resection of non-functioning adrenal adenomas: influencing factors and serum metabolic features

**DOI:** 10.3389/fmolb.2025.1524121

**Published:** 2025-02-13

**Authors:** Jianlei Zhang, Peiqiang Wu, Yule Chen, Min Wang, Wenbin Song

**Affiliations:** Department of Urology, The First Affiliated Hospital of Xi’an Jiaotong University, Xi’an, Shaanxi, China

**Keywords:** non-functioning adrenal adenomas (NFAs), hypertension, metabolomics, guanidoacetic acid (GAA), influencing factors

## Abstract

**Introduction:**

Non-functioning adrenal adenomas (NFAs) are typically regarded as benign tumors that lack hormonal secretion. However, emerging evidence has shown that some patients with NFAs and hypertension experience improvements in blood pressure after adrenalectomy, indicating a potential correlation between NFAs and hypertension. Nevertheless, the precise mechanisms that underpin this phenomenon remain elusive.

**Methods:**

We collected data from all patients with adrenal adenomas who underwent unilateral laparoscopic partial or total adrenalectomy at the First Affiliated Hospital of Xi’an Jiaotong University in 2023. A statistical analysis was conducted on factors such as tumor diameter, duration of hypertension, BMI, and age. Additionally, we gathered serum samples from six patients who experienced postoperative blood pressure improvement and six patients who did not. These samples were subjected to targeted and untargeted metabolomic analyses to identify significant serum metabolites.

**Results:**

Our findings revealed that 50.9% of patients with NFAs and hypertension experienced blood pressure improvement after surgery. Additionally, patients in the improvement group (IG) exhibited larger tumor diameters alongside shorter durations of hypertension compared to their counterparts in the nonimprovement group (NIG). Untargeted metabolomic analysis identified 89 differentially abundant metabolites in the serum between the IG and NIG. In particular, we found that guanidinoacetic acid (GAA), a precursor of creatine synthesis that possibly participates in the occurrence of hypertension, was enriched in patients in the IG and reduced after surgery.

**Discussion:**

The findings of our study indicated that duration of hypertension and tumor diameter may exert an influence on the extent of postoperative blood pressure improvement, and NFAs might promote hypertension through GAA-related creatine metabolism.

## 1 Introduction

Adrenal adenomas are defined as tumours that arise in the adrenal cortex. Based on their endocrine function, adrenal adenomas can be categorized into four main types, i.e., aldosterone-producing adenomas, sex hormone-producing adenomas, cortisol-producing adenomas, and nonfunctioning adenomas (NFAs) (Mete et al., 2022). Among them, NFAs are characterized as adrenal tumor that do not produce excess hormones. In contrast to functional adenomas that secrete hormones such as cortisol and aldosterone, NFAs are distinguished by their lack of hormonal activity. Typically identified incidentally through imaging studies, NFAs are generally considered benign lesions (Fassnacht et al., 2016; Terzolo et al., 2005; Zeiger et al., 2009). As evidenced in the pertinent literature, NFAs are the most common type of adrenal tumor, constituting almost 80% of the cases (Cho et al., 2013; Akkuş et al., 2018; Goh et al., 2018). Although NFAs are not associated with overt hormonal secretion, approximately 20∼30% of patients with NFAs are found to have concomitant hypertension, and 40% of them experience hypertension improvement after surgery (Bancos et al., 2016). Nevertheless, the precise mechanisms that underpin this phenomenon remain elusive.

In this work, the investigation was conducted for the patients with adrenal adenomas who have undergone adrenal surgery within the past year, with a particular focus on the relationship between NFAs and hypertension improvement. Our study aims to investigate the contributing factors to patients with NFAs and hypertension and to characterize their serum metabolic profiles using both untargeted and targeted metabolomics. Our findings revealed that postoperative blood pressure improvement was associated with tumor diameter and duration of hypertension. Furthermore, serum metabolomics analysis was employed to detect differentially abundant metabolites in NFA patients with or without hypertension improvement. A total of 89 metabolites were identified as being differentially enriched between the improvement group (IG) and the nonimprovement group (NIG) groups with regard to blood pressure following adrenalectomy. These included metabolites associated with cardiovascular disease, obesity, and metabolic disorders, such as arachidonoylserotonin, L-propionylcarnitine, and guanidinoacetic acid.

## 2 Materials and methods

### 2.1 Patients

The data set comprised all patients with adrenal adenomas who underwent unilateral laparoscopic partial or complete adrenalectomy at the First Affiliated Hospital of Xi’an Jiaotong University, within the Department of Urology, in 2023. The investigation comprised 205 cases with an average interval of 3 weeks between adrenal adenoma diagnosis and surgery, among which 91 were diagnosed with primary aldosteronism (PA), 25 with Cushing’s syndrome (CS), and 89 with NFAs. These patients were diagnosed with unilateral adrenal cortical adenoma through enhanced computed tomography, and all were detected as adrenal adenomas by postoperative pathological examination. The data set comprised patients’ demographic characteristics (e.g., age, height, weight), duration of hypertension, preoperative and postoperative blood pressure, and blood levels of adrenal-related hormones (e.g., aldosterone, renin, ARR, cortisol, catecholamines and their metabolites in the blood). In the metabolomics study, the serum samples of 12 patients were obtained both preoperatively and postoperatively. The patient cohort included six individuals who exhibited improved blood pressure following surgery and six patients who did not. All participants provided informed consent prior to their involvement.

### 2.2 Blood pressure improvement standard

Patients’ blood pressure was monitored 1 month after adrenal surgery. Three blood pressure measurements were taken from each patient, preceded by a 5-min rest period for each measurement. The average of these three readings constituted the patient’s blood pressure. On the basis of the postoperative blood pressure changes, patients with NFAs and combined hypertension were divided into the IG and the NIG groups. The term “blood pressure improvement” was defined as follows. First, blood pressure returned to normal without the use of antihypertensive medication; Second, blood pressure control was improved by using the same antihypertensive medication as before the operation; Third, blood pressure could be effectively controlled by using a smaller amount of antihypertensive medication. The main types of oral antihypertensive drugs taken by the patients were commonly seen, including nifedipine, amlodipine, irbesartan, and valsartan. Blood pressure improvement was defined as meeting at least one of three specified criteria. In contrast, the nonimprovement of blood pressure was considered if there was no improvement or minimal improvement in blood pressure, as well as if there was no change or increase in the type or dosage of antihypertensive medicine required to control blood pressure.

### 2.3 Serum collection

Patients were phlebotomized 1 day prior to surgery and 3 days after surgery on an empty stomach. The peripheral blood samples were collected, incubated at 4°C for 4 h until coagulation, and then subjected to centrifugation at 2,000 rpm. The collected supernatant was preserved at −80°C prior to metabolomics analysis. Ultimately, six IG and six NIG serum samples were were used to analyze the differential serum metabolites between the two patient groups.

### 2.4 Metabolite extraction

Untargeted metabolomics analysis was performed on pre-operative serum samples from the IG and NIG groups. Samples were slowly thawed at 4°C. An appropriate volume was then added to a pre freezing mixture of water/methanol/acetonitrile (1:2:2, v/v) and vortexed. The mixture was first sonicated at low temperature for 30 min. It was then incubated at −20°C for 10 min. The supernatant was collected after centrifugation at 14,000 g for 20 min at 4°C, and then vacuum-dried. The dried sample was prepared for mass spectrometry analysis by adding 100 µL of a water/acetonitrile solution (1:1, v/v). The mixture was then vortexed and centrifuged at 14,000 g for 15 min at 4°C. Collected for analysis, the supernatant was ready for further investigation.

### 2.5 LC–MS analysis

The Agilent 1,290 Infinity LC UHPLC system (Agilent Technologies) was employed for metabolomics analysis. The chromatographic column used was the Waters ACQUITY UPLC BEH Amide 1.7 μm, 2.1 mm × 100 mm column. Aqueous solutions of 25 mM CH₃COONH₄ and 25 mM NH₄OH (designated as solvent A), in addition to acetonitrile (designated as solvent B) was employed as mobile phase. Gradient elution was conducted by initiating the process with 95% solvent B for 0.5 min, subsequently reducing to 65% over 6.5 min, then to 40% in 1 min. After kept for 1 min, the concentration is rapidly increased to 95%, followed by a 3-min equilibrium period. The flow rate was 0.4 mL/min. The electrospray ionization (ESI) source were established with ion source gas 1 and 2 (60), curtain gas (30), source temperature at 600°C, and an ion spray voltage float of ±5500 V. The m/z range in pure mass spectrometry (MS) mode is set to 60–1,000 Da with a spectrum accumulation time of 0.20 s. The m/z range in automatic MS/MS mode was set to 25–1,000 Da with a spectrum accumulation time of 0.05 s. Product ion scans were performed in high sensitivity information-dependent acquisition. The collision energy (CE) was maintained at 35 V ± 15 eV, with declustering potentials of +60 V for positive ions and −60 V for negative ions. Isotopes differing by a mass of 4 Da were ignored. At least 10 ions were detected during each acquisition cycle.

### 2.6 Data processing and statistical analysis

Metabolites were identified using an in-house database (Shanghai Applied Protein Technology). ProteoWizard MSConvert was utilized for transforming the raw mass spectrometry data into MzXML files, which was then analyzed with the aid of XCMS software. The peak picking was performed utilizing the centWave algorithm with m/z of 10 ppm, a peak width within 10–60 s, and a prefilter threshold at 10 ions per 100 intensity units. The parameters employed during peak grouping were as follows, such as the bandwidth (bw) at 5 s, the m/z width (mzwid) of 0.025 Da, and the minimum fraction (minfrac) of 0.5. Isotopes and adducts were identified with the aid of the CAMERA tool. Ion with a minimum of 50% nonzero measurements were kept for the subsequent analysis. The identification of metabolites is achieved by comparing the m/z values (within 10 ppm) and MS/MS spectra against a comprehensive internal reference standard database. After the standardized processing of the data, the R package “ropls” was applied for analysis, since it integrates Pareto-scaled principal component analysis and orthogonal partial least squares discriminant analysis. To guarantee the accuracy of model, 7-fold cross-validation was performed, along with response permutation testing. Furthermore, variable importance in projection (VIP) scores were computed to assess the contribution of each variable to the classification. For comparisons between IGs and NIGs, the p-value was determined by either a t-test, applicable for normally distributed data, or a Wilcoxon signed-rank test, suitable for non-normally distributed data. In the OPLS-DA, metabolites were considered significantly different if they possessed a VIP score above 1 and a p-value below 0.05. The metabolites having VIP values above 1 and p-values between 0.05 and 0.1 were categorized as differentially abundant metabolites, as their differences were nearly statistically significant (Carrillo et al., 2016). Volcano plots were generated using the ggplot2 package in R to visualize differentially abundant metabolites. The x-axis represents the log2-transformed fold change, and the y-axis represents the negative logarithm (base 10) of the p-value [-log10 (p-value)]. Furthermore, Pearson correlation analysis was utilized to evaluate the interrelationships among the variables.

Baseline data (e.g., age and tumor diameter) of patients were subjected to analysis via GraphPad Prism 8.0. In instances where a continuous variable fails to adhere to a normal distribution, the data are presented as medians accompanied by interquartile ranges. A two-sample independent t-test was employed for the purpose of comparing two groups of data. In the case of count data, a chi-square test was employed for the purpose of making comparisons between groups. Finally, for nonnormally distributed continuous data, the Wilcoxon rank-sum test was employed to evaluate the differences. Logistic regression analysis was performed using SPSS to assess the relationship between postoperative blood pressure improvement and age, tumor diameter, and hypertension duration. OR > 1 indicated an unfavorable factor for postoperative blood pressure improvement, while an OR < 1 indicated a favorable factor. A p-value of less than 0.05 is indicative of a significant difference.

### 2.7 HPLC-UV was used for GAA quantification on serum samples

Targeted metabolomics was used to quantify serum GAA levels pre- and post-surgery in the IG and NIG groups. Quantification of GAA was carried out using Agilent OpenLab CDS software with a calibration curve generated from standards. The x-axis represents the standard concentration, and the y-axis represents the peak area. GAA standard solutions were prepared at concentrations of 0.1 mg/mL, 0.2 mg/mL, and 0.4 mg/mL. Limit of Detection (LOD) was set at 0.01 mg/mL, and Limit of Quantification (LOQ) was set at 0.033 mg/mL. Serum samples (0.5 mL) were diluted to 4 mL, vortexed for 2 min, centrifuged at high speed for 10 min, and the supernatant was filtered prior to analysis. An Agilent 1,260 system was employed for detecting the guanidinoacetic acid concentration by using Hypersil-NH2 column (4.6 mm × 250 mm, 5 μm), a mobile phase of acetonitrile–0.02 moL KH2PO4 aqueous (PH = 8) and then gradient elution, and the subsequent filtration using a membrane with a pore size of 0.45 μm. The analysis was detected at 210 nm, with an injection volume of 10 μL and a flow rate of 1.0 mL/min. The metabolites were subjected to quantitative analysis.

## 3 Results

### 3.1 Clinical characteristics of the participants

**FIGURE 1:**
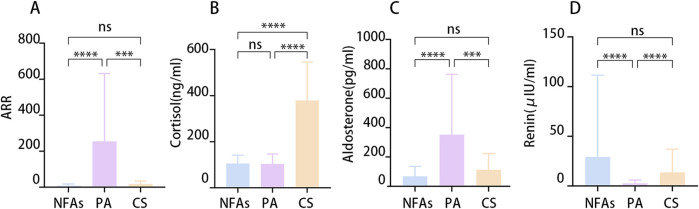
The differential hormonal characteristics between the groups. **(A)** Aldosterone-renin ratio (ARR). **(B)** Serum Cortisol at 8:00 a.m. **(C)** Concentration of Aldosterone. **(D)** Concentration of Renin. (*p < 0.05).

205 patients with adrenal adenoma underwent unilateral laparoscopic adrenal surgery in 2023. Ninety-one (44.39%) patients were diagnosed with primary aldosteronism, 25 (12.2%) were diagnosed with Cushing’s syndrome, and 89 were diagnosed with NFA. Fifty-three (59.55%) patients with NFAs had hypertension. Table 1 presents the demographic characteristics in detail. A test of adrenal hormones revealed that patients with primary aldosteronism exhibited elevated aldosterone levels and ARRs, while displaying lower renin concentrations compared to patients with NFA or Cushing’s syndrome. Conversely, patients with Cushing’s syndrome demonstrated higher cortisol levels than patients with NFA or primary aldosteronism did (Figure 1).

**TABLE 1:** The demographic characteristics of the patients.

	PA (n = 91)	NFAs(n = 89)	CS(n = 25)
BMI	24.57 ± 2.8	25.2 ± 3.19	24.63 ± 2.91
Age years	47.95 ± 12.53	53.66 ± 12.16	38.36 ± 2.07
Gender			
Male	38	43	11
Female	53	46	14
Hypertension			
Yes	91	53	18
No	0	36	7

### 3.2 Influencing factors of blood pressure improvement

**FIGURE 2:**
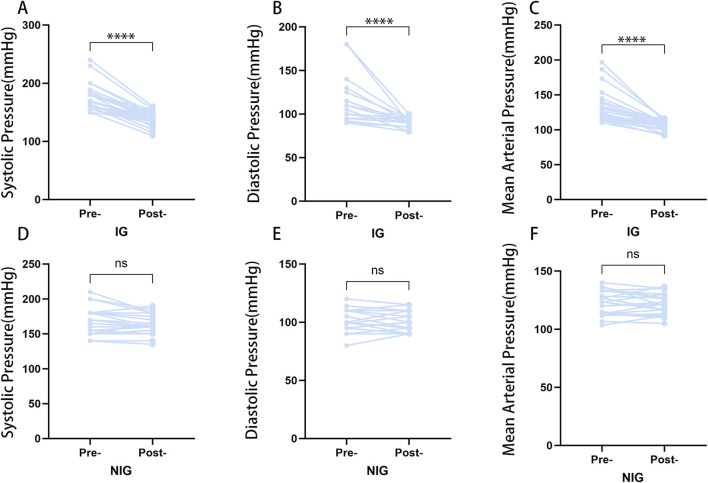
Postoperative blood pressure changes in the IG and NIG. (*p < 0.05) **(A)** Changes in systolic blood pressure in the IG group. **(B)** Changes in diastolic blood pressure in the IG group. **(C)** Changes in mean arterial pressure in the IG group. **(D)** Changes in systolic blood pressure in the NIG group. **(E)** Changes in diastolic blood pressure in the NIG group. **(F)** Changes in mean arterial pressure in the NIG group.

Among patients with NFAs caused by hypertension, 27 patients (50.9%) exhibited an improvement in blood pressure 1 month following surgery.The patients with NFAs and hypertension were divided according to their blood pressure after surgery into the IG (n = 27) and the NIG (n = 26). The IG demonstrated significant improvements in postoperative systolic, diastolic, and mean arterial pressure, whereas the NIG did not exhibit any notable changes (Figure 2).

The relationships between clinicopathological characteristics and postoperative blood pressure improvement was subjected to further analysis. The findings revealed no statistically significant differences between the IG and NIG groups with regard to gender, BMI, or preoperative blood pressure between the IG and NIG. However, patients in the IG group was younger, and had a larger tumor diameter and a shorter duration of hypertension compared to those in the NIG group, with a p-value of less than 0.05 (Table 2).

**TABLE 2:** Demographic characteristics of patients in IG and NIG.

	IG (n = 27)	NIG (n = 26)	P
Age years(y)	49.5 ± 2.1	58 ± 2.3	0.009
BMI	25.8 ± 0.6	25.5 ± 0.6	0.73
Tumor diameter(cm)	2.1 ± 0.1	1.8 ± 0.1	0.045
Duration of hypertension(y)	4 ± 0.9	10.5 ± 1.7	0.001
Gender			0.487
Male	14	11	
Female	13	15	
Types of drugs	1.3 ± 1.9	1.6 ± 1.4	0.193

**FIGURE 3:**
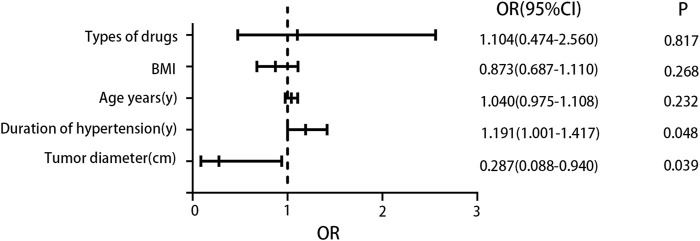
Logistic regression analysis results. The results indicated that a larger tumor diameter was a favorable factor for blood pressure improvement, while a longer duration of hypertension was an unfavorable factor for blood pressure improvement.

We performed logistic regression analysis to investigate the relationship between various factors (including patient age, duration of hypertension, types of drugs, BMI, and tumor diameter) and postoperative blood pressure improvement. The findings revealed that both tumor diameter and duration of hypertension were significantly correlated with postoperative blood pressure improvement. Specifically, a shorter duration of hypertension and a larger tumor diameter were associated with a greater likelihood of improved blood pressure after surgery. For a detailed presentation of the analysis results, please refer to Figure 3.

The clinical characteristics of the 12 patients who underwent untargeted metabolomics analysis were analyzed, and no statistically significant differences were found. The specific results are presented in Table 3.

**TABLE 3:** Demographic characteristics of 12 non targeted metabolomics patients.

	IG (n = 6)	NIG (n = 6)	P
Age years(y)	53.2 ± 2.8	55.2 ± 5	0.735
BMI	25.4 ± 1.5	25.2 ± 1.3	0.407
Tumor diameter(cm)	2.1 ± 0.2	2.3 ± 0.2	0.398
Duration of hypertension(y)	5.7 ± 2.1	4.4 ± 2.2	0.679
Gender			0.999
Male	4	3	
Female	2	3	
Types of drugs	1.2 ± 0.3	1.3 ± 0.2	0.664

### 3.3 Differentially abundant metabolites between IG and NIG patients

**FIGURE 4:**
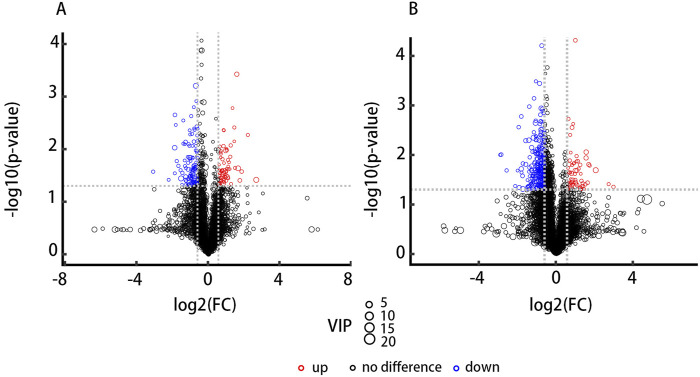
Volcano plots depict the results of differential abundance analysis. (FC) > 1.5 or <0.67 and p-value <0.05. The x-axis represents the log₂-transformed fold change, and the y-axis represents the -log₁₀-transformed p-value. Significantly upregulated metabolites are shown in red, significantly downregulated metabolites in blue, and non-significant metabolites in black. **(A)** The positive ion-mode results. **(B)** The negative ion-mode results.

A total of 523 known positive ion-mode biochemical compounds and 485 negative ion-mode compounds were detected from nontargeted metabolomic analysis. A total of 89 differential metabolites were detected, positive ion mode revealed 11 metabolites with significantly different abundances (p < 0.05, VIP>1) and an additional 30 metabolites exhibiting differential abundance (0.05 ≤ p < 0.1, VIP>1). Negative ion mode showed significant abundance differences in 16 metabolites and differential abundance in 32 metabolites. Table 4 lists the significantly differential metabolites. Univariate analysis was performed on all detected metabolites (including unidentified metabolites) in both positive and negative ion-modes to identify differentially abundant metabolites. Volcano plots (Figure 4) illustrate metabolites exhibiting a fold change.

**TABLE 4:** Details of significant differential metabolites. Significantly upregulated metabolites are shown in red, significantly downregulated metabolites in blue.

Positive ion mode				Negative ion mode			
Name	VIP	Fold change	p-value	Name	VIP	Fold change	p-value
Pro-Trp	3.65	0.83	0.00	Cis,cis-muconic acid	3.49	0.89	0.03
3,5,9-trioxa-4-phosphatetracosan-1-aminium, 7-(acetyloxy)- 24-carboxy-4-hydroxy-n,n,n-trimethyl-, inner salt, 4-oxide, (r)-	3.28	0.61	0.00	Pi 34:2	3.39	0.49	0.02
1-myristoyl-sn-glycero-3-phosphocholine	2.97	0.52	0.05	Pi(18:1/13-hode)	3.03	0.48	0.03
Val-Ala-Lys	2.86	0.67	0.01	Dehydrotumulosic acid	2.89	0.72	0.05
L-propionylcarnitine	2.61	0.54	0.03	3.beta.,7.alpha.acid	2.56	0.64	0.05
Mitoxantrone	2.52	0.36	0.04	Valine	2.35	0.74	0.02
N-myristoylsphinganine	2.06	0.90	0.01	Arachidonoylserotonin	2.21	0.71	0.02
1,2-diamino-2-methylpropane	2.00	0.77	0.03	Roburic acid	2.19	0.74	0.03
Dimethyl sulfone	1.92	0.83	0.04	Phenylethyl2-glucoside	1.86	0.47	0.02
Olmesartan medoxomil	1.53	0.42	0.04	Geldanamycin	1.57	0.49	0.03
Arachidoyl ethanolamide	1.32	0.87	0.01	Arachidic acid	1.50	0.74	0.02
				Pi 37:4	1.34	0.59	0.02
				Pi 36:2	1.31	0.71	0.02
				L-Isoleucine	1.19	0.62	0.02
				Celastrol	1.14	0.57	0.02
				Guanidoacetic acid	1.12	1.99	0.00

**FIGURE 5:**
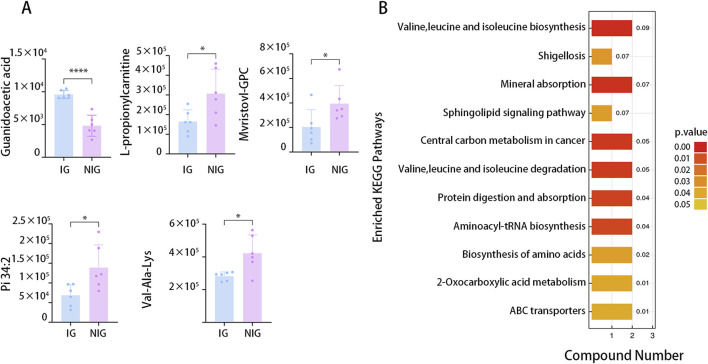
Differential metabolites between IG and NIG. **(A)** Top five differential serum metabolites detected by LC–MS analysis in IG and NIG. **(B)** KEGG analysis of the differential metabolites detected by LC–MS analysis in IG and NIG. (*P < 0.05).

The results obtained in positive and negative ion modes revealed that, in comparison to the NIG, the IG exhibited 18metabolites with greater abundance and 71 metabolites with lower abundance. The greatest fold change was observed for GAA, which was 2-fold greater in the IG, while Pi (18:1/13-hode) demonstrated the greatest fold change (2.1-fold greater) in the NIG (Figure 5A).

To further investigate the potential biological pathways associated with the differentially expressed metabolites, we conducted KEGG enrichment analysis. The analysis included all the different metabolites identified in all the ion modes. The results indicated the enrichment of 11 pathways, including those engaged in mineral absorption, the internal carbon metabolism, as well as aminoacyl-tRNA biosynthesis (Figure 5B).

### 3.4 HPLC analysis of the guanidinoacetic acid concentration

**FIGURE 6:**
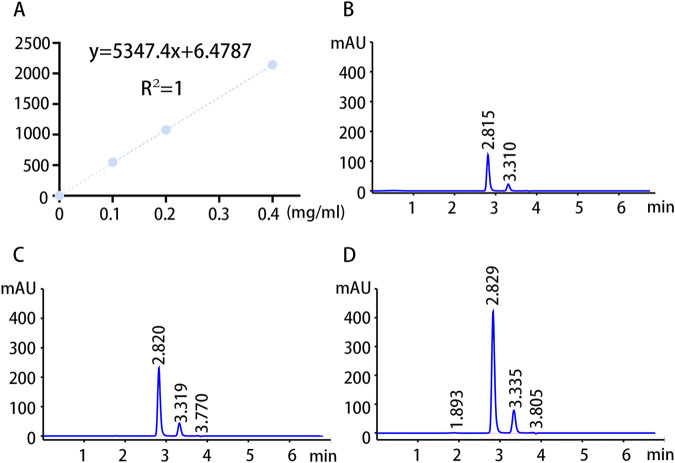
Standard curves and chromatograms of targeted metabolomics standards. **(A)** Targeted metabolomics standard curves. **(B)** Chromatogram of GAA standard at a concentration of 0.1 mg/mL. **(C)** Chromatogram of GAA standard at a concentration of 0.2 mg/mL. **(D)** Chromatogram of GAA standard at a concentration of 0.4 mg/mL.

**FIGURE 7:**
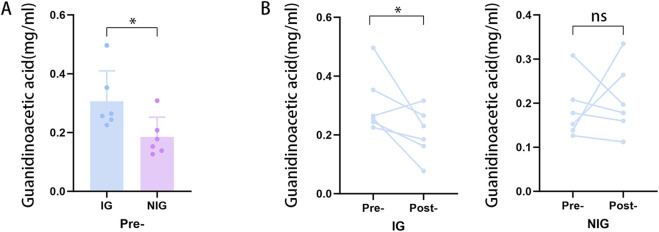
Targeted metabolomics analysis results. **(A)** Preoperative differences in GAA in the IG and NIG. (*p < 0.05) **(B)** Pre- and postoperative changes of GAA in IG and NIG. (*p < 0.1).

To validate our findings, the concentration of GAA in pre- and postoperative serum samples from patients with NFAs was detected through HPLC analysis using targeted metabolomics analysis. The standard curves and chromatograms of the standards for targeted metabolomics are shown in Figure 6. The results of the metabolomic analysis were corroborated by the finding of obviously elevated GAA concentration in IG compared to NIG (Figure 7A). Furthermore, a notable decline in GAA levels was observed in the IG group following surgical intervention, whereas minimal alterations were evident in the NIG group (Figure 7B). These findings indicate that GAA may have a potential role in blood pressure improvement after surgery in patients with NFAs.

### 3.5 Correlation analysis of GAA levels with clinical characteristics in NFA patients

**FIGURE 8:**
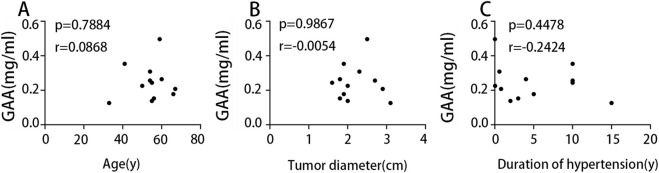
**(A)** Correlation analysis of age and GAA. **(B)** Correlation analysis of tumor diameter and GAA. **(C)** Correlation analysis of duration of hypertension and GAA.

To investigate the influence of factors such as age on the metabolomic findings, correlation analyses were performed to assess the association between serum GAA levels and these factors. Results (Figure 8) indicated no significant correlation between serum GAA levels and age, tumor diameter, or hypertension duration. This suggests that GAA may independently influence blood pressure in NFA patients.

## 4 Discussion

Adrenal tumors are responsible for approximately 3%–10% of all cases of hypertension (Vaidya et al., 2018), making adrenal disease a significant contributor to secondary hypertension. Although there is definitive evidence that adrenal adenomas causing primary aldosteronism and Cushing’s syndrome are responsible for secondary hypertension, some studies and clinical observations have demonstrated that some patients with nonfunctioning adrenal adenomas may experience an improvement in blood pressure after surgical resection (Bancos et al., 2016). Furthermore, these adenoma patients frequently present with a range of metabolic issues (Ribeiro et al., 2018). Nonetheless, the fundamental mechanisms have yet to be thoroughly investigated. Thus, this work aimed to investigate the factors influencing the improvement of postoperative blood pressure through a retrospective analysis and a metabolomic approach.

In our retrospective study group, 50.9% of patients with NFAs and hypertension exhibited an improvement in postoperative blood pressure, which aligns with the reported work (Bancos et al., 2016). Our analysis indicated a significant correlation between postoperative blood pressure improvement and a number of factors, including tumor diameter and hypertension duration. In particular, the larger the tumour, the higher the rate of blood pressure improvement. Conversely, the longer hypertension duration,the lower the probability of blood pressure improvement. These finding matched up with the report by Xu et al., in which longer hypertension duration of more than 6 years were identified as risk factors for suboptimal blood pressure improvement after surgery (Xu et al., 2015). Our findings similarly indicate that tumor diameter and hypertension duration are critical factors influencing the improvement status of patients with NFAs and hypertension postoperatively.

Nontargeted metabonomic analysis demonstrated a great variance in GAA levels between the IG and NIG. This finding was subsequently validated by HPLC analysis. Further observations indicated a pronounced reduction in the concentration of GAA in the IG group after surgery, whereas there was no significant change in the concentration of GAA in the NIG group. This suggests that the levels of GAA can be hypothesized as a biomarker to assess whether patients with NFA and hypertension will experience blood pressure improvement after surgery. GAA is a precursor for creatine synthesis, and participates in the creatine metabolic pathway (Ostojic and Jorga, 2023). The synthesis of creatine from GAA necessitates the consumption of arginine, which may decrease the availability of nitric oxide (Jahangir et al., 2009), potentially resulting in elevated blood pressure. Furthermore, Mohd Saleh Ahmad Kamal et al. have proposed that the creatine metabolic pathway may exert an influence on blood pressure (Kamal et al., 2022). Positive correlation between plasma creatine levels and the risk of hypertension in men was reported in several studies (Post et al., 2022). It was therefore hypothesized that NFAs might impact blood pressure through GAA-related creatine metabolism pathway. Correlation analysis of GAA with age, hypertension duration, and tumor diameter revealed no significant associations. This suggests that GAA may be an independent factor influencing blood pressure.

Significant differences were found in arachidonic acid, serotonin, as well as L-propionylcarnitine between IG and NIG patients among these metabolites. These substances exert a pivotal role in the pathogenesis of inflammatory conditions, obesity, diabetes, hypercholesterolemia, and cardiovascular diseases (Mingorance et al., 2011a; Mingorance et al., 2011b; Scioli et al., 2015; Sonnweber et al., 2018). These findings suggest that these metabolites might be potential factors contributing to adrenal NFA-associated hypertension. We also observed differential levels of arachidonoyl ethanolamide (AEA), an endogenous fatty acid amide and component of the endocannabinoid system (ECS) (Lu and Mackie, 2016), between the IG and NIG groups. Given the ECS’s established role in hypertension pathogenesis (Golosova et al., 2022), this suggests a potential involvement of AEA in the abnormal blood pressure observed in NFA patients.

Untargeted metabolomics analysis also identified substances not synthesized in humans, such as geldanamycin and mitoxantrone. However, analysis of the patient’s diet and recent medication history revealed no exposure to these compounds. These false positives likely arose from the inherent susceptibility to false discoveries in metabolomics-based biomarker discovery (Zhang et al., 2021). We will conduct further research to better annotate those species flagged as drugs by the software, as they clearly share m/z values and chromatographic parameters.

The KEGG analysis of the differential metabolites between IG and NIG patients identified several enriched pathways, including those related to mineral absorption, central carbon metabolism in cancer, protein digestion, and aminoacyl-tRNA biosynthesis, indicating a broad impact of adrenal non-functioning adenoma on metabolism. This could also suggest that NFAs possess a potential secretory or metabolic function.

## 5 Conclusion

The present study observed an improvement in blood pressure following adrenalectomy in a subset of patients with NFA. The potential influencing factors for postoperative blood pressure improvement included duration of hypertension and tumour diameter. Additionally, alterations in serum metabolites were detected between the IG and NIG groups. Notably, patients in the IG exhibited elevated level of GAA, which underwent a postoperative reduction. GAA is a precursor in the synthesis of creatine and possibly participates in the occurrence of hypertension, indicating that NFAs might contribute to hypertension via GAA metabolism. Further studies are essential to confirm the findings as well as elucidate the molecular mechanisms involved. This also suggests that the levels of GAA can be hypothesized as a biomarker to assess whether patients with NFA and hypertension will experience blood pressure improvement after surgery. Our approach is exploratory, therefore, all findings require further validation in future studies.

## Data Availability

The raw data supporting the conclusions of this article will be made available by the authors, without undue reservation.
